# Radiosynthesis and Evaluation of Talazoparib and Its Derivatives as PARP-1-Targeting Agents

**DOI:** 10.3390/biomedicines9050565

**Published:** 2021-05-18

**Authors:** Dong Zhou, Huaping Chen, Cedric Mpoy, Sadia Afrin, Buck E. Rogers, Joel R. Garbow, John A. Katzenellenbogen, Jinbin Xu

**Affiliations:** 1 Department of Radiology, School of Medicine, Washington University in Saint Louis, Saint Louis, MO 63110, USA; chenhuaping@wustl.edu (H.C.); sadia@wustl.edu (S.A.); garbow@wustl.edu (J.R.G.); 2 Department of Radiation Oncology, School of Medicine, Washington University in Saint Louis, Saint Louis, MO 63110, USA; cmpoy@wustl.edu (C.M.); b.rogers@wustl.edu (B.E.R.); 3 Department of Chemistry, University of Illinois at Urbana-Champaign, Urbana, IL 61801, USA; jkatzene@illinois.edu; 4 Cancer Center at Illinois, University of Illinois at Urbana-Champaign, Urbana, IL 61801, USA

**Keywords:** PARP-1, Talazoparib, binding assay, biodistribution, targeted radiotherapy

## Abstract

Poly (ADP-ribose) polymerase-1 (PARP-1) is a critical enzyme in the DNA repair process and the target of several FDA-approved inhibitors. Several of these inhibitors have been radiolabeled for non-invasive imaging of PARP-1 expression or targeted radiotherapy of PARP-1 expressing tumors. In particular, derivatives of olaparib and rucaparib, which have reduced trapping potency by PARP-1 compared to talazoparib, have been radiolabeled for these purposes. Here, we report the first radiosynthesis of [^18^F]talazoparib and its in vitro and in vivo evaluation. Talazoparib (**3a″**) and its bromo- or iodo-derivatives were synthesized as racemic mixtures (**3a**, **3b** and **3c**), and these compounds exhibit high affinity to PARP-1 (*K*_i_ for talazoparib (**3a″**): 0.65 ± 0.07 nM; **3a**: 2.37 ± 0.56 nM; **3b**: 1.92 ± 0.41 nM; **3c**: 1.73 ± 0.43 nM; known PARP-1 inhibitor Olaparib: 1.87 ± 0.10 nM; non-PARP-1 compound Raclopride: >20,000 nM) in a competitive binding assay using a tritium-labeled PARP-1 radioligand [^3^H]WC-DZ for screening. [^18^F]Talazoparib (**3a″**) was radiosynthesized via a multiple-step procedure with good radiochemical and chiral purities (98%) and high molar activity (28 GBq/μmol). The preliminary biodistribution studies in the murine PC-3 tumor model showed that [^18^F]talazoparib had a good level of tumor uptake that persisted for over 8 h (3.78 ± 0.55 %ID/gram at 4 h and 4.52 ± 0.32 %ID/gram at 8 h). These studies show the potential for the bromo- and iodo- derivatives for PARP-1 targeted radiotherapy studies using therapeutic radionuclides.

## 1. Introduction

**Figure 1:**
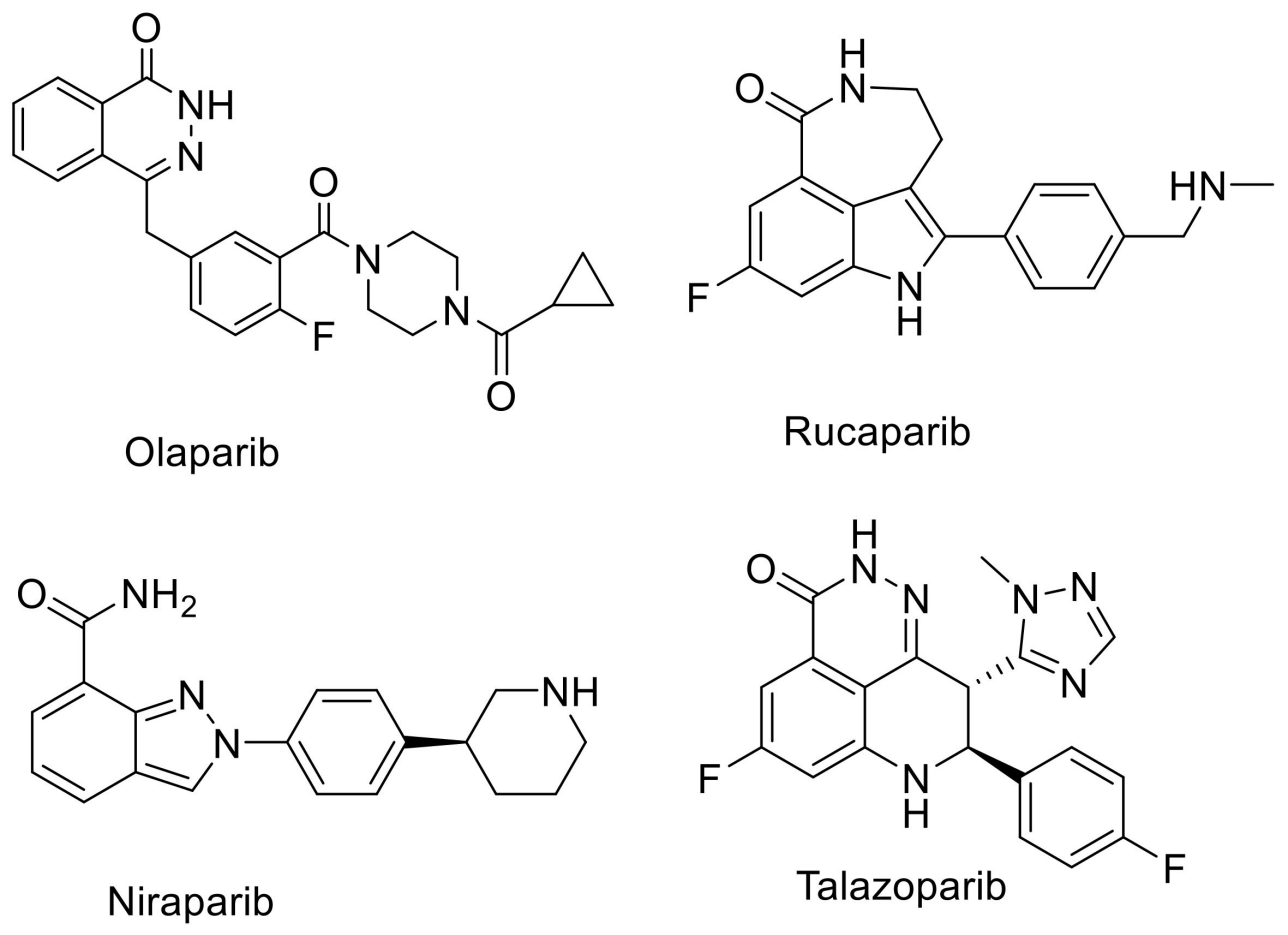
PARP-1 inhibitors approved by US FDA for oncology use.

Poly (ADP-ribose) polymerase-1 (PARP-1), an abundant and ubiquitous enzyme, predominantly associates with chromatin in the nucleus and participates in DNA repair and regulation of gene expression [1,2]. During DNA repair, PARP-1 catalyzes ADP-ribose (PAR) moieties and polymers attachment covalently onto itself and other engaged nuclear proteins via PARylation with the donor molecule, nicotinamide adenine dinucleotide (NAD^+^). PARP-1 senses DNA damage by binding to the site of DNA single-strand breaks (SSBs) and initiating auto-PARylation to repair SSBs; after repair, PAR is degraded. PARP-2 is also involved in DNA repair by the same mechanism as PARP-1, but PARP-1 is more active than PARP-2 and catalyzes nearly 90% of the PAR formation in cell [3]. PARP-1 has been investigated as a therapeutic oncology target [4], it induces synthetic lethality efficiently in cancers that are deficient in certain DNA repair mechanisms (BRCA1/2 and ATM) and dependent on upregulated PARP-1 activity for survival [5]. Over the past decade, many PARP-1 inhibitors have been reported [4], and the FDA has approved olaparib, rucaparib, niraparib, and talazoparib (Figure 1) for the intervention of a variety of cancers.

As an important oncology and imaging target, several PARP-1 nuclear imaging agents, based on olaparib and rucaparib, have been reported [6], including [^18^F]FluorThanatrace ([^18^F]FTT) [7] and [^18^F]FPARPi [8], which are in clinical trials. Pre-clinical and clinical studies have demonstrated that radiolabeled PARP-1 ligands are capable of measuring PARP expression in tumors [9], quantifying the efficacy of several FDA-approved PARP-1 inhibitors in lung cancer in vitro and in vivo [10], and monitoring PARP-1 expression in response to radiation therapy [11]. Because of the mechanism of PARP-1 inhibition, PARP-1 inhibitors have also been labeled with therapeutic radioisotopes (e.g., At-211 [12], Br-77 [13], I-123 [14], I-125 [15], and I-131 [16]) to deliver radiation directly to the site of SSBs where the radiation will cause further DNA damage, resulting in DNA double-strand breaks (DSBs) and eventually cell death [17].

Most PARP inhibitors mimic the NAD+ substrate of PARP-1, bind to the catalytic domain of PARP-1 and inhibit PARylation [18]. PARP inhibitors, however, may show differential abilities to trap PARP-1/2 at the site of DNA damage. Comparatively, talazoparib is ~100 fold more efficient in PARP trapping than olaparib [19]. This high trapping of PARP inhibitors prevents the dissociation of PARP from the SSBs of DNA and thus enables a longer retention of PARP radioligands at SSB sites, improving the potential of delivering radiotherapy with longer-lived radionuclides. Furthermore, talazoparib contains fluorine atoms for radiolabeling with F-18 without structural modification. 

The objective of this project was to prepare and evaluate talazoparib and its derivatives as radioligands for PARP-1 targeted radiotherapy. We have synthesized talazoparib and its bromo- and iodo-derivatives and evaluated their PARP-1 inhibition potencies and binding affinities. We also radiosynthesized [^18^F]talazoparib and evaluated its tumor-specific retention in a murine model (PC-3 prostate cancer) that we previously used for the evaluation of PARP-1 radioligands. 

## 2. Method and General Information

### 2.1. General Information

All chemicals purchased from commercial vendors were used without further characterization or purification. Unless otherwise stated, the organic synthesis reactions were conducted under an inert atmosphere with dry solvents using standard air-free and moisture-free techniques. Carrier free [^18^F]fluoride was generated by the ^18^O(p, n)^18^F reaction via proton irradiation of ^18^O enriched water (95%) using a RDS111 or ACSI TR-19 cyclotron. High-performance liquid chromatography (HPLC) was performed with an ultraviolet detector and a well-scintillation NaI (Tl) detector and associated electronics for radioactivity detection. Radio-TLC was accomplished using a Bioscan AR-2000 imaging scanner (Bioscan, Inc., Washington, DC). Published methods were used for the synthesis of compounds **1**, **2a**, **3a** [20], and **4** [21], and the same procedures [20] were used to make **2b**, **2c**, **3b**, and **3c**, as shown below.

### 2.2. Synthesis of Methyl 2-(4-bromophenyl)-7-fluoro-3-(1-methyl-1H-1,2,4-triazol-5-yl)-4-oxo-1,2,3,4-tetrahydroquinoline-5-carboxylate (2b) and Methyl 7-fluoro-2-(4-iodophenyl)-3-(1-methyl-1H-1,2,4-triazol-5-yl)-4-oxo-1,2,3,4-tetrahydroquinoline-5-carboxylate (**2c**) 

Compound **2b** was synthesized following the same procedure as **2a**. Starting from methyl 5-fluoro-2-(2-(1-methyl-1H-1,2,4-triazol-5-yl)acetyl)-3-nitrobenzoate (**1**) (0.5 g, 1.55 mmol), 4-bromobenzaldehyde (0.6 g, 3.24 mmol), and 30% TiCl_3_(III) in 2N HCl (8 mL)—**2b** (0.67 g) was obtained via flash chromatography (silica gel/1:1 ethyl acetate/hexanes) as a yellowish solid. ^1^H NMR (400 MHz, CDCl_3_) δ (ppm) 7.75 (s, 1H), 7.39–7.37 (m, 2H), 7.24–7.21 (m, 2H), 6.48–6.44 (m, 2H), 5.25 (d, *J* = 12.8 Hz, 1H), 5.08 (s, 1H), 4.12 (d, *J* = 12.8 Hz, 1H), 3.82 (s, 3H), 3.53 (s, 3H); ^13^C NMR (100 MHz, CDCl_3_) δ (ppm) 186.02, 168.95, 168.92, 167.98, 165.42, 153.01, 152.97, 150.44, 149.67,138.48, 138.37, 137.21, 132.08, 128.99, 123.09, 111.37, 111.39, 107.06, 106.82, 103.06, 102.81, 60.36, 53.01, 51.82, 35.16; HRMS: calcd. for C20H17BrFN4O3 [M+H]^+^ 459.0463; found 459.0446.

Compound **2c** was synthesized following the same procedure as **2a**. Starting from **1** (0.5 g, 1.55 mmol), 4-iodobenzaldehyde (0.6 g, 2.59 mmol) and 30% TiCl_3_(III) in 2N HCl (8 mL), **2c** (0.59 g) was obtained via flash chromatography (silica gel/1:1 ethyl acetate/hexanes) as a yellowish solid. ^1^H NMR (400 MHz, CDCl_3_) δ (ppm) 7.75 (s, 1H), 7.57 (d, *J* = 8.4 Hz, 2H), 7.09 (d, *J* = 8.0 Hz, 2H), 6.46–6.44 (m, 2H), 5.22 (d, *J* = 12.8 Hz, 1H), 5.11 (s, 1H), 4.12 (d, *J* = 12.8 Hz, 1H), 3.82 (s, 3H), 3.53 (s, 3H); ^13^C NMR (100 MHz, CDCl_3_) δ (ppm) 185.99, 168.95, 168.92, 165.42, 153.10, 152.97, 150.42, 149.67, 138.46, 138.35, 138.01, 137.91, 129.17, 111.34, 107.01, 106.76, 103.06, 102.81, 94.82, 60.40, 53.00, 51.74, 35.17; HRMS: calcd. for C20H17FIN4O3 [M+H]^+^ 507.0324; found 507.0310.

### 2.3. Synthesis of 8-(4-bromophenyl)-5-fluoro-9-(1-methyl-1H-1,2,4-triazol-5-yl)-2,7,8,9-tetrahydro-3H-pyrido[4,3,2-de]phthalazin-3-one (3b) and 8-(4-iodophenyl)-5-fluoro-9-(1-methyl-1H-1,2,4-triazol-5-yl)-2,7,8,9-tetrahydro-3H-pyrido[4,3,2-de]phthalazin-3-one (**3c**)

Compound **3b** was synthesized following the same procedure as **3a**. Starting from **2b** (0.60 g, 1.31 mmol) and hydrazine monohydrate (0.5 mL), **3b** (0.53 g) was obtained as a white solid (M.P. 312–314 °C). ^1^H NMR (400 MHz, DMSO-*d_6_*) δ (ppm) 12.33 (s, 1H), 7.78 (s, 1H), 7.69 (s, 1H), 7.51–7.48(m, 2H), 7.39–7.36 (m, 2H), 7.04 (dd, *J* = 2.2, 9.0 Hz, 1H), 6.88 (dd, *J* = 2.2, 11.0 Hz, 1H), 5.00–4.94 (m, 2H), 3.65 (s, 3H); ^13^C NMR (100 MHz, DMSO-*d*_6_) δ (ppm) 166.59, 164.12, 159.30, 152.31, 150.75, 148.70, 141.35, 138.99, 131.73, 130.71, 130.30, 121.92, 111.72, 103.39, 103.13, 99.14, 98.90, 59.07, 42.76, 35.35; HRMS: calcd. for C19H15BrFN6O [M+H]^+^ 441.0469; found 441.0459.

Compound **3c** was synthesized following the same procedure as **3a**. Starting from **2c** (0.59 g, 1.17 mmol) and hydrazine monohydrate (0.5 mL), **3c** (0.38 g) was obtained as a white solid (M.P. 315–317 °C). ^1^H NMR (400 MHz, DMSO-*d_6_*) δ (ppm) 12.32 (s, 1H), 7.77 (s, 1H), 7.68–7.67 (m, 2H), 7.65 (s, 1H), 7.23–7.21 (m, 2H), 7.03 (dd, *J* = 2.4, 8.8 Hz, 1H), 6.87 (dd, J = 2.4, 11.2 Hz, 1H), 4.98–4.91 (m, 2H), 3.65 (s, 3H); ^13^C NMR (100 MHz, DMSO-*d_6_*) δ (ppm) 166.59, 164.12, 159.30, 152.33, 150.74, 148.70, 141.36, 139.36, 137.59, 130.78, 130.30, 111.71, 103.37, 103.10, 99.11, 98.87, 95.07, 59.20, 42.70, 35.35; HRMS: calcd. for C_19_H_15_FIN_6_O [M+H]^+^ 489.0331; found 489.0334.

### 2.4. Synthesis of [^18^F]Talazoparib (**3a″**)

A solution of [^18^F]TsF (47 mCi) in acetonitrile (1 mL) was added into a 10 mL Pyrex tube containing precursor **4** (2 mg, 6.4 µmol) and pre-dried potassium carbonate/Kryptofix^®^ 222 (2 mg, 2.2 µmol). The tube was capped and then heated at 108 °C for 8 min. RadioTLC indicated a 97% radiochemical conversion to [^18^F]**5**. At room temperature, a solution of compound **1** (2.9 mg, 9.0 µmol) in methanol (0.5 mL) and a solution of 20% TiCl_3_ in 2 N HCl (0.5 mL) were added to the above reaction. The reaction was then heated at 60 °C for 7 min to convert [^18^F]**5** to [^18^F]**2a** completely, according to radio-HPLC. The reaction mixture was diluted with water to 8 mL and the diluted mixture passed through an HLB plus cartridge. The cartridge was rinsed with water (15 mL), 40.8 mCi radioactivity was trapped in the cartridge and 1.5 mCi was eluted in water. The cartridge was first eluted with methanol (0.6 mL), this eluted portion (containing 12 mCi) was discarded as the following step is slow in the presence of excess water. The remaining radioactivity was eluted with methanol (3 × 0.5 mL). NH_2_NH_2_·H_2_O (100 µL) was added into the eluted methanol solution (26.4 mCi) and the mixture was heated at 108 °C for 15 min to complete the reaction. The mixture was diluted with water (3 mL) for semi-HPLC purification (Column: Phenomenex Luna C18 250 × 10 mm; Mobile phase: 30% acetonitrile/70% water/0.1% TFA; Flow rate: 4 mL/min; UV: 254 nm). The desired product, [^18^F]**3a**, was collected at 18 min (11.7 mCi) and diluted with water (40 mL). The diluted solution passed through an HLB light cartridge, which was rinsed with water (5 mL) afterward. The radioactivity was eluted with methanol in portion (0.1 mL) to afford 11.2 mCi. The methanol was removed at 108 °C under argon flow, and the residue (10.2 mCi) was dissolved in methanol (40 µL) and water (60 µL). This solution (8 mCi) was injected for chiral separation using an analytical HPLC (Column: Chirobiotech T 250 × 4.6 mm; Mobile phase: 40% methanol/60% 0.05 M ammonium formate butter (pH = 4.5); Flow rate: 1 mL/min; UV: 254 nm). Compound [^18^F]**3a’** was collected at 9.8–10.6 min and compound [^18^F]**3a″;** ([^18^F]talazoparib) (3.17 mCi) was collected at 11.8–12.6 min. The [^18^F]**3a″** collected was diluted with water (10 mL) and passed through an HLB light cartridge which was subsequently rinsed with water (5 mL). The final [^18^F]**3a″** product was eluted with ethanol in portion (0.1 mL) and the portion containing the most radioactivity was diluted with saline (0.9 mL) to prepare the dose for animal studies (1.56 mCi in 1000 µL 10% ethanol/90% saline). The animal dose was analyzed under the same conditions for chiral separation to determine chiral and radiochemical purities and molar activity.

### 2.5. Xenograft Model

The animal studies were approved by Washington University’s Institutional Animal Care and Use Committee (IACUC), Protocol #20-0214 approved 7 August 2020. The overall animal care was following The Guide for the Care and Use of Laboratory Animals from the National Research Council and the USDA Animal Care Resource Guide. Mature SCID mice from Charles River Laboratories were allowed to acclimate in an AALAC accredited housing facility for at least 1 week before tumor implantation for the biodistribution study. Male SCID mice (4 weeks old, *n* = 4/group, 2 groups), with 1 × 10^7^ PC-3 prostate cancer cells implanted subcutaneously in the right shoulder grew for about 35 days before the radiotracer biodistribution studies.

### 2.6. Biodistribution

Animals were intravenously injected with [^18^F]talazoparib (120 µCi) in 10% ethanol/saline (100 µL) via the tail vein. At the specified time point (4 and 8 h), the mice were sacrificed, the blood, lungs, liver, spleen, kidneys, muscle, fat, heart, brain, bone, bone marrow, prostate, pancreas, and tumor were harvested, weighed, and counted in a γ-counter. The percentage injected dose per gram of tissue (%ID/g) was determined by decay correction of the [^18^F]talazoparib for each sample and then normalized by the standard injected radiotracer dose and the sample weight.

### 2.7. Materials and Cell Culture

The standard compounds (Raclopride and Olaparib) and other chemical reagents were purchased from Sigma-Aldrich (St. Louis, MO, USA) and Tocris (Ellisville, MO, USA). The stock solutions of compounds at 3mM were dissolved in N, N-Dimethylformamide (DMF), dimethyl sulfoxide (DMSO), or ethanol and then diluted to RPMI 1640 medium to achieve different concentrations for cell-uptake based binding assays. [^3^H]WC-DZ was custom synthesized by ViTrax (Placentia, CA, USA) from a bromo-precursor (WC-DZ-Br) that was synthesized and supplied by our group (Figure S1).

U251MG cells (RRID: CVCL_0021) were grown in RPMI 1640 medium (Invitrogen, Carlsbad, CA, USA) supplemented with 10% fetal bovine serum (Invitrogen, Carlsbad, CA, USA) and 1% penicillin/streptomycin (Invitrogen, Carlsbad, CA, USA). Cells were maintained in a humidified environment of 5% CO_2_ and 95% air at 37 °C. The annually authentication for quality and integrity assurance via short tandem repeat (STR) profiling was serviced by the University of Arizona Genetics Core as we have reported [22].

### 2.8. Radioligand Cell Uptake Saturation Analyses

U251MG cell saturation uptake assays with [^3^H]WC-DZ (0.5–15 nM) were carried out in 96-well tissue culture plates (Fisher Scientific, Pittsburgh, PA, USA), 150 µL of media per well, at 37 °C for 30 min, then washed with 200 µL of ice-cold PBS. And each individual well was transferred and counted in a scintillation vial with 2 mL counting fluid using a liquid scintillation counter (Hitachi Aloka Medical, Ltd, Tokyo, Japan) to measure the cell-bound radioactivity. Nonspecific binding was defined using the wells with 10 µM Olaparib to block the PARP-1 sites in the U251MG cells.

The dissociation constant (*K*_d_) at equilibrium and the maximum binding density (*B*_max_) were determined using the Scatchard linear fitting method [23].

The Hill coefficient, *n*_H_, as shown in the following equation transformed from the saturation cell uptake binding:

$$\log\frac{B_{s}}{B_{\max}-B_{s}}=\log K_{d}+n_{H}\log L$$

where *L* stands for the radioligand concentration. *n*_H_, Hill slope, was derived by a linear regression analysis of plotting of $\log\frac{B_{s}}{B_{\max}-B_{s}}$ vs. logL.

### 2.9. Competitive Assay

U251MG cells (~16,000 cells per well) were seeded the day before the assay and incubated in a total volume of 150 µL of media with the radioligand at 37 °C in 96 well plates for 30 min. The final concentration of the radioligand in each assay was 6 nM. Compound (Talazoparib, **3a**, **3b**, **3c**, olaparib, raclopride) concentrations ranging from 0.1 nM to 1 µM were added to acquire the inhibition curves. After the reaction was completed, the cells were washed once with ice-cold PBS and the bound radioactivity was counted and analyzed as described above. Nonspecific binding was determined from cells treated with 10 µM Olaparib.

The nonlinear regression analysis was used to model the competitive binding data to calculate the *IC*_50_ value, the concentration of an inhibitor that inhibits 50% of the radioligand specific binding using a single-site binding equation:

Bs=B0−B0∗IIC50+I

where, *B*_s_ stands for the specific bound (i.e., *B*_s_ = *B*_t_ − *B*_ns_, where *B*_t_ is the total and *B*_ns_ is the nonspecific bound radioactivity of the radiotracer), *B*_0_ is the bound amount in the absence of the competitive inhibitor, *I* stands for the inhibitor concentration. Competitive dissociation constants (*K_i_* values) were derived from the Cheng and Prussoff equation [24]:

Ki=IC501+LtKd

In which the *K_d_* values of [^3^H]WC-DZ (6.71 ± 1.24 nM) were obtained from U251MG saturation experiments and *L_t_* is the radioligand concentration of the assay.

Data from competitive binding were transformed to determine the pseudo-Hill coefficient, *n′*_H_, using the following equation:

$$\log\frac{B_{s}}{B_{0}-B_{s}}={n^{\prime }}_{H}\log I-{n^{\prime }}_{H}\log IC_{50},$$

in which the negative of the Hill slope, *n′*_H_, can be readily calculated by a linear regression of plotting $\log\frac{B_{s}}{B_{0}-B_{s}}$ vs. logI, as we published [22,25].

## 3. Results and Discussion

### 3.1. Organic Synthesis and Radiosynthesis

**Scheme 1:**
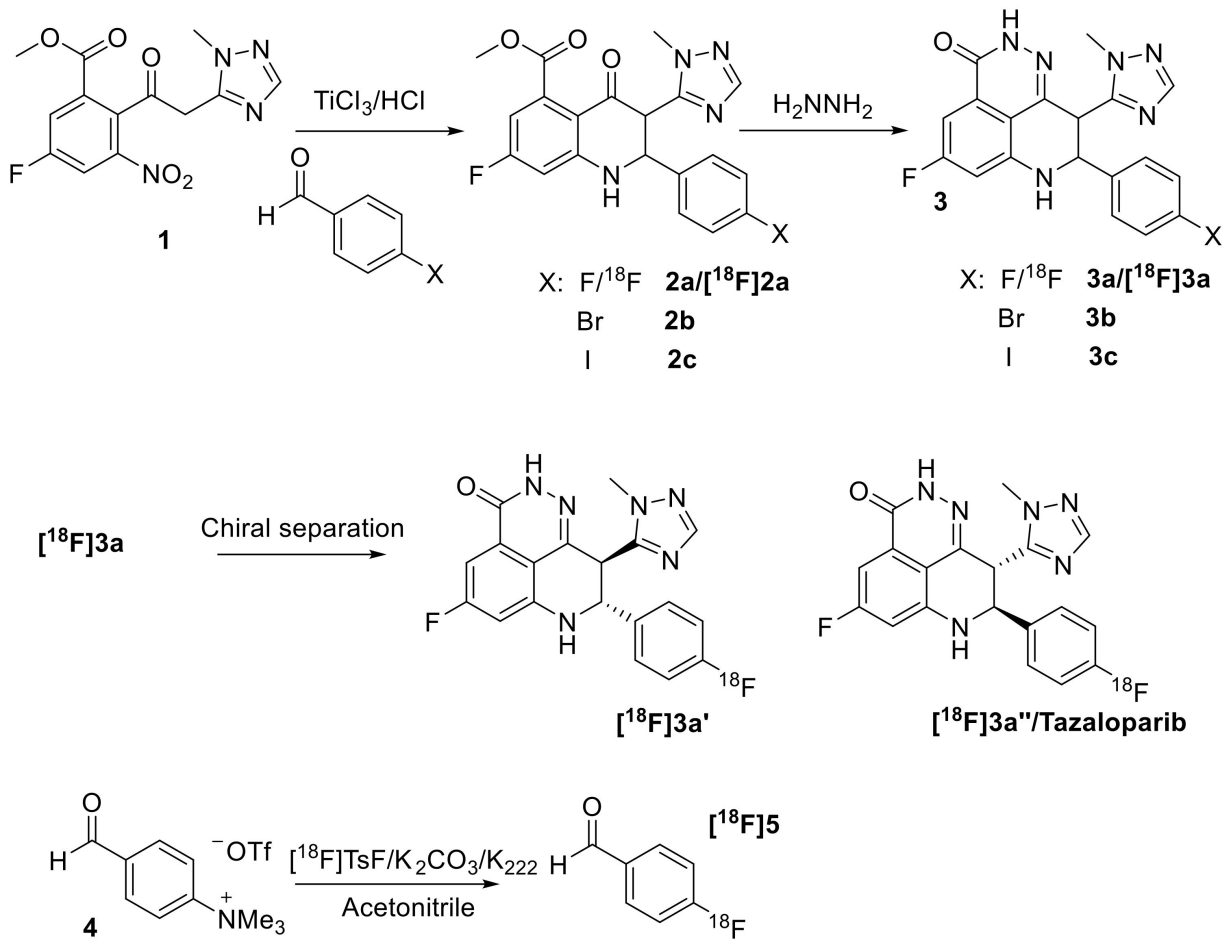
Synthesis of talazoparib and its derivatives and radiosynthesis of [^18^F]talazoparib/**3a″**.

Published procedures were followed to synthesize talazoparib (**3a″**) and its bromo- and iodo-derivatives (Scheme 1) [20]. Starting from **1**, each step was completed in a short time and with a high yield to afford the final products, **3a–c**. According to chiral HPLC, two isomers were obtained for compound **3a–c** (Figure S2), which is consistent with the literature [20]. The late-eluted isomer of the racemic mixture **3a** in the chiral HPLC was confirmed as talazoparib by co-injection of the authentic compound. According to the literature [20], the two isomers of **3b** and **3c** are expected to be the thermodynamically more favorable trans-isomers. However, we were not able to separate the two enantiomers in large quantities using our HPLC chiral column. In the literature, supercritical fluid chromatography (SFC) was used for the chiral separation using supercritical carbon dioxide and methanol as the mobile phase. The racemic mixture was used for binding assay.

For the radiosynthesis of [^18^F]talazoparib (**3a″**) we chose the reported multiple-step procedures for the synthesis of **3a** instead of using a late-stage radiofluorination strategy because the reported reactions are fast and high yielding, which allowed the radiosynthesis to be carried out promptly and in high yield. The direct radiosynthesis of [^18^F]talazoparib (**3a″**) using the currently available strategy requires considerable effort for the synthesis of proper precursors and testing of the labeling step. Therefore, [^18^F]talazoparib ([^18^F]**3a″**) was synthesized from compound **1** and [^18^F]4-fluorobenzaldehyde ([^18^F]**5**) following the same procedure used to make talazoparib. [^18^F]**2a** was formed as the major radioactive product in the one-pot reaction under mild reaction conditions. However, the addition of H_2_NNH_2_ directly to the above reaction mixture failed to afford any [^18^F]**3**. After standard SPE purification of [^18^F]**2** and discarding the first elution portion, which contained most of the water from SPE, [^18^F]**2a** was consumed quickly, and [^18^F]**3a** was collected in a 63% decay corrected yield in 44 min (Figure S3). Chiral separation of [^18^F]**3a** afforded [^18^F]talazoparib (**3a″**) in 98% chiral and radiochemical purities with molar activity being 28.1 GBq/µmol at the end of synthesis (Figure S4). The identity of [^18^F]talazoparib was confirmed by chiral HPLC co-injection of authentic talazoparib (Figure S5).

### 3.2. Synthesis of [^3^H]WC-DZ

[^3^H]WC-DZ was prepared by ViTrax (Placentia, CA) via a direct catalytic tritium gas exchange with the Br atom of WC-DZ-Br (Figure S1). WC-DZ (as reference) [26] and WC-DZ-Br [27] were synthesized according to the published scheme. The radiochemical purity was determined by HPLC to be >99%. Product identity confirmation was examined by mass spectrometry and HPLC co-elution with the unlabeled WC-DZ (Retention time: 10 min; Column: Zorbax RX-C18, 4.6 × 150 mm, 5 µm; Gradient elution: mobile phase A (0.05% TFA/water) and mobile phase B (acetonitrile, from 10% B to 90% B over 10 min at flow rate 1 mL/min.). The molar activity, 0.85 GBq/mmol (23.1 Ci/mmol), was measured by mass spectrometry.

### 3.3. Saturation Analyses of Radioligand Uptake in U251MG Cells

**Figure 2:**
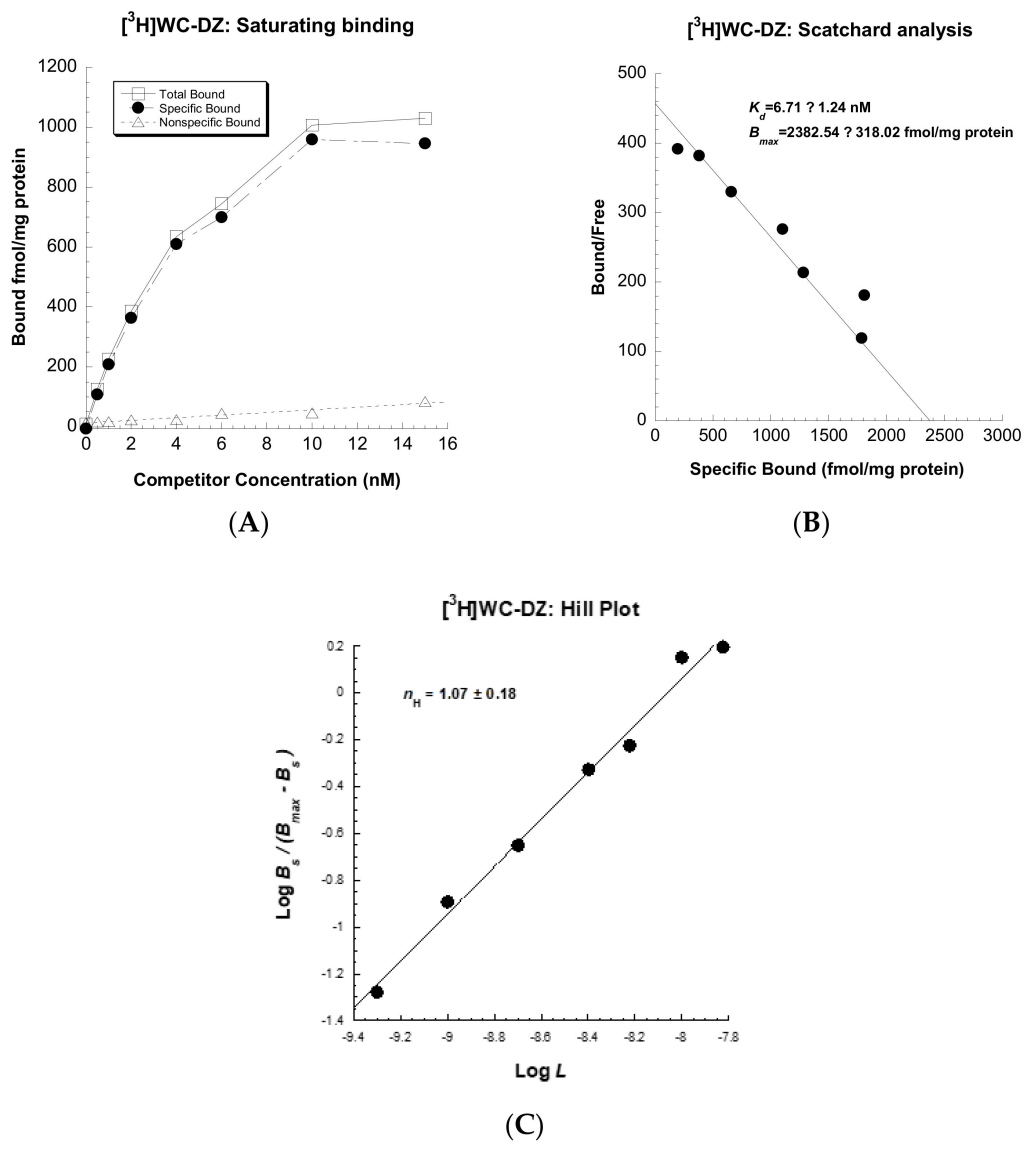
[^3^H]WC-DZ binding to PARP-1 in U251MG cells. (**A**) Saturation binding curves show the total, non-specific, and specific bound respectively. (**B**) Scatchard plot analysis was used to derive the *K_d_* and *B_max_* values: *K_d_* = 6.71 ± 1.24 nM, *B_max_* = 2382.54. ± 318.02 fmol/mg protein. (**C**) Hill plot was used to determine the Hill coefficient: *n*_H_ = 1.07 ± 0.18. *n* = 3, samples in triplicate. Mean ± SEM.

To characterize WC-DZ (with *K*_i_ =4.1 nM against human full-length PARP-1) [26] as a potential radiotracer for screening PARP-1 inhibitors, its affinity to PARP-1 was first determined in live cells. The saturation binding with increasing concentrations of [^3^H]WC-DZ were carried out in U251MG cells. The saturation curve, Scatchard analysss, and Hill plots are presented in Figure 2. [^3^H]WC-DZ has a dissociate constant value of 6.71 ± 1.24 nM (*K*_d_), a binding density value of 2382 ± 318 fmol/mg protein (*B*_max_), and a Hill plot value of 1.07 ± 0.18 (*n*_H_) for U251MG cells. Taken collectively, U251MG cell line is reliable ezyme resource for screening PARP-1 inhibitors, using [^3^H]WC-DZ as the radiotracer ligand.

### 3.4. Competitive Profile of Standard and WC-DZ in U251MG Cells

**Figure 3:**
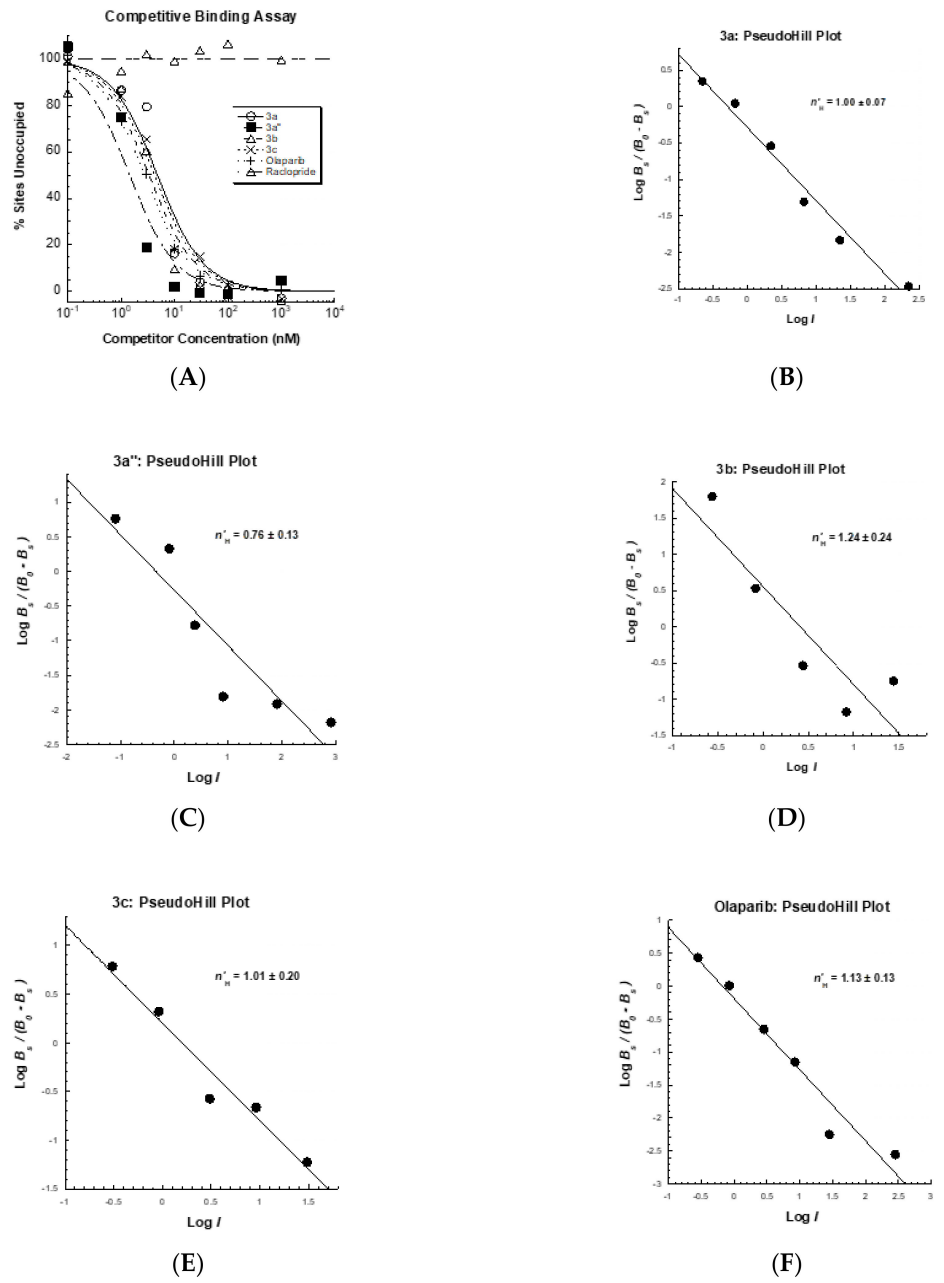
Competitive binding for inhibition of the [^3^H]WC-DZ binding to PARP-1 in glioblastoma cells (U251MG) by **3a**, **3a″**, **3b**, **3c**, olaparib, and raclopride—a known PARP-1 nonselective compound. (**A**) Representative competitive binding data *K*i. **3a**: 2.37 ± 0.56 nM, **3a″**: 0.65 ± 0.07 nM, **3b**: 1.92 ± 0.41 nM, **3c**: 1.73 ± 0.43 nM, olaparib: 1.87 ± 0.10 nM, raclopride: >20,000 nM. (**B**) (**3a**), (**C**) (3a″), (**D**) (**3b**), (**E**) (**3c**) and (**F**) (olaparib): Representative pseudo Hill plots for determining the pseudoHill coefficient (*n′*_H_ values). **3a**: *n’*_H_ = 1.00 ± 0.07; **3a″**: *n′*_H_ = 0.76 ± 0.13; **3b**: *n′*_H_ = 1.24 ± 0.24; **3c**: *n′*_H_ = 1.01 ± 0.20; Olaparib: *n’*_H_ = 1.13 ± 0.13. *n* = 3, samples in triplicate. Mean ± SEM.

To screen for PARP-1 inhibitors, competitive binding assays were performed using reported and novel PARP-1 inhibitors, which include a well-studied PARP-1 inhibitor-olaparib, talazoparib, and its derivatives, and WC-DZ. These assays show that **3a″** (the authentic talazoparib compound) has a high binding affinity, with a *K*_i_ of 0.65 ± 0.07 nM, while olaparib shows a *K*_i_ of 1.87 ± 0.10 nM, indicating that its affinity is lower than that of talazoparib. **3a**, **3b**, and **3c**, as racemic mixtures, also show a high affinity to PARP-1 at the same level as olaparib (Table 1). According to the literature, one enantiomer in each pair has much better in vitro activity than the other enantiomer [20]; so, we expect that the same would be true for the bromo- and iodo-derivatives of talazoparib **3b** and **3c** and that one of the enantiomers of **3b** and **3b** would have binding affinity comparable to talazoparib. No apparent displacement was detected for raclopride—a nonselective compound (Figure 3A). The values of the pseudoHill coefficient, *n′*_H_, are 1.00 ± 0.07, 0.76 ± 0.13, 1.24 ± 0.24, 1.01 ± 0.20 and 1.13 ± 0.13 for **3a**, **3a″**, **3b**, **3c**, and olaparib, respectively, (Figure 3B–F) and are summarized in Table 1.

### 3.5. Biodistribution Studies of [^18^F]Talazoparib

**Figure 4:**
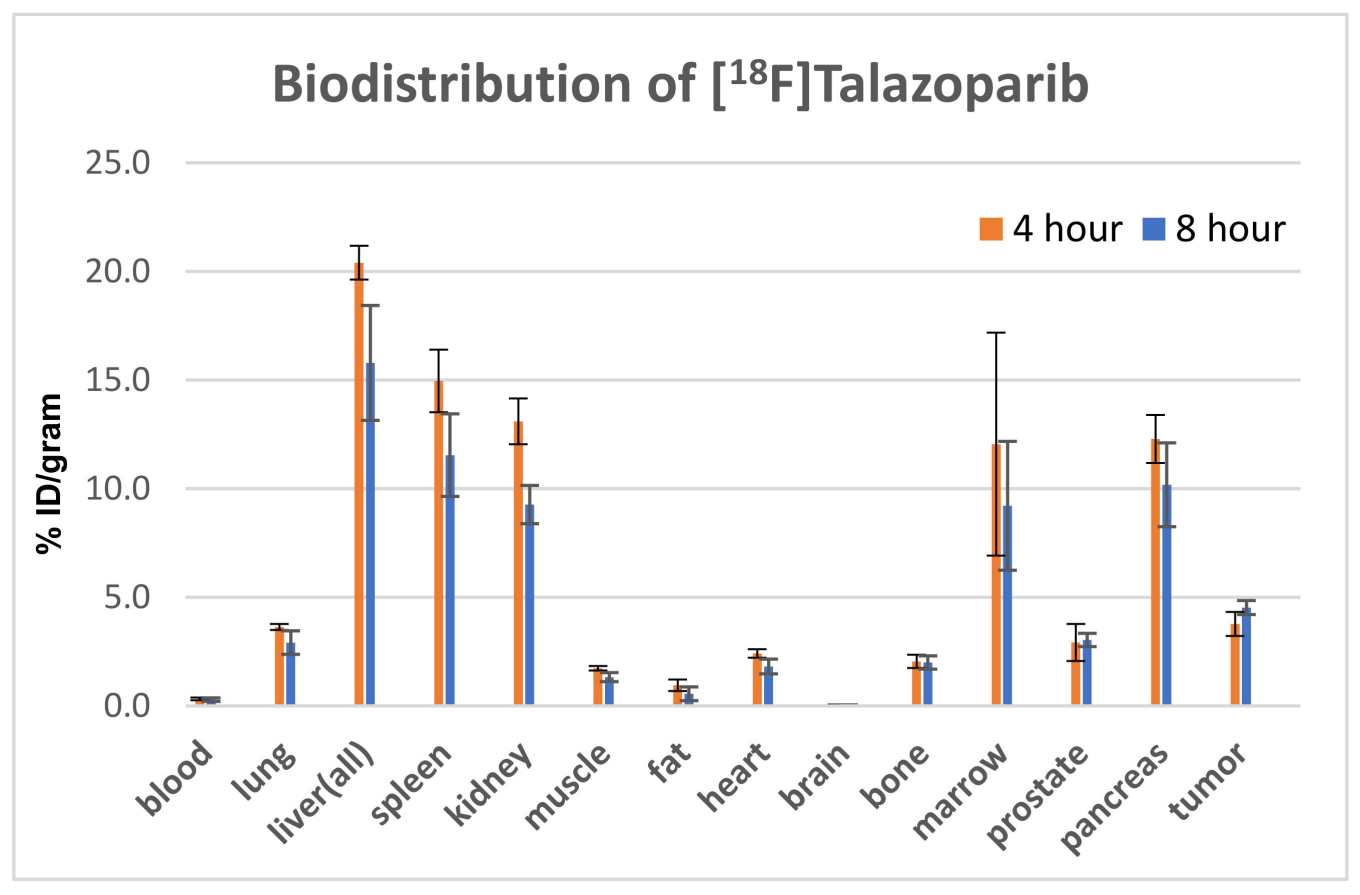
Biodistribution of [^18^F]talazoparib/**3a″** in mature SCID mice with PC-3 prostate tumors (4 per group) at 4 and 8 h post-injection.

The retention and trapping of [^18^F]talazoparib in tumors was determined by a biodistribution study in PC-3 tumor-bearing mice, assessing tissue activity levels at prolonged time points (4 and 8 h), as shown in Figure 4. Our group has used the PC-3 prostate cancer tumor model to validate several PARP-1 radioligands, including [^18^F]WC-DZ-F [28]. [^18^F]talazoparib had good uptake at 4 h and was retained in the PC-3 tumors for over 8 h (3.78 ± 0.55 % ID/gram at 4 h and 4.52 ± 0.32 %ID/gram at 8 h). The retention of [^18^F]talazoparib in PC-3 tumors is much longer than that of the radioiodine-labeled olaparib derivatives [29,30] (e.g., 0.5 ± 0.09 %ID/g at 1 h and 0.17 ± 0.06 %ID/g at 2 h in U87 MG tumor-bearing mice) [30]. Even though no data is available for the retention of radiolabeled rucaparib derivatives-[^18^F]FTT [7] and [^125^I]KX1 [31] in tumors over 2 h, the fast clearance of radioactivity from spleens after 2 h and before 4 h indicates that the retention of radioactivity in targets will be less than 4 h. The long retention of [^18^F]talazoparib in the tumors is consistent with literature reports: (1) Talazoparib significantly attenuated intratumoral PAR concentration at 2 and 8 h time points, with a partial recovery at 24 h [20]; (2) Pretreatment with talazoparib can block PARP-1 radioligand uptake up to 24 h before the administration of the radioligand [32]. Radioactivity was cleared from the blood within 4 h. High uptake of [^18^F]talazoparib, however, was observed in the liver, spleen, kidney, and pancreas after 4 h and was only slightly reduced at 8 h. In general, high uptake in the above organs was commonly observed at early times for previously reported PARP-1 radioligands but quickly washed out after 2 h. Since talazoparib has been reported as a substrate of P-glycoprotein (P-gp) [33], brain uptake was extremely low (0.05 ± 0.01 %ID/gram at 4 h), which is consistent with other reports [34]. The uptake in bone marrow is high at 4 and 8 h, and bone marrow toxicity is reported for talazoparib. This may be a concern for PARP-1 targeted radiotherapy and should be taken into account in future study design.

Talazoparib is an FDA-approved drug targeting PARP-1. It is stable and specific for PARP-1 and has great trapping potency [20]. Although the trapping mechanisms and the structural basis for PARP-1 retention are more complicated as proposed [35], the biodistribution of [^18^F]talazoparib generally agrees with the published data on talazoparib. Its bromo- and iodo-derivatives with suitable molecular weight and lipophilicity have comparable potency towards PARP-1. Once labeled with therapeutic radioisotopes, these halogen analogs may have a better potential for PARP-1 targeted radiotherapy and may merit further investigation.

## 4. Conclusions

PARP-1 radiotracers with high affinity and selectivity, and appropriate in vivo pharmacologic properties, can serve as robust biomarkers for monitoring cancer progression, metastasis, and evaluating PARP-1 related oncologic interventions. Radioligand therapy using PARP-1 radiotracers can deliver radiation directly to DNA and induce effective cell death without damaging surrounding somatic cells that have no PARP-1 expression.

In this communication, a PARP-1 inhibitor WC-DZ was synthesized and radiolabeled with tritium. A novel radioactive binding assay using [^3^H]WC-DZ in a PARP-1 high expression glioblastoma cell line was validated and set up for in vitro measurement of PARP-1 in cancer tissues or cell lines and for high throughput screening of next-generation PARP-1 inhibitors.

Talazoparib and its bromo- or iodo-derivatives were prepared and their high binding affinities (<2 nM) were determined by competing with [^3^H]WC-DZ. [^18^F]talazoparib was also radiosynthesized with high specific activity and preliminary biodistributions studies were conducted. [^18^F]talazoparib tumor retention in the PC-3 tumor is high and persists to late time points (4 and 8 h). The high uptake and extended retention of [^18^F]talazoparib in the tumors are in agreement with its reported high trapping efficiency. It will be of great interest to examine this radiotracer and radiolabeled bromo- and iodo-derivatives and test these PARP-1 radiotracers in different PARP-1-enriched cancer models to develop more potent radiotracers with favorable in vivo properties, using talazoparib as a leading scaffold.

## Figures and Tables

**Table 1:** Talazoparib and its bromo- and iodo-derivatives as PARP-1 inhibitors.

	3a″ ^a^	3a	3b	3c	Olaparib	Raclopride
*K*_i_ (nM) ^b^	0.65 ± 0.07	2.37 ± 0.56	1.92 ± 0.41	1.73 ± 0.43	1.87 ± 0.10	>20,000
*n*′_H_	0.76 ± 0.13	1.00 ± 0.07	1.24 ± 0.24	1.01 ± 0.20	1.13 ± 0.13	None
cLogP ^c^	2.58	2.58	3.25	3.78	/	/
MW	380.36	380.36	441.26	488.26	/	/

^a^ Authentic compound (talazoparib); ^b^ Mean ± SEM; ^c^ Calculated by Chemdraw Pro 16.

## Data Availability

All data have been included in this communication.

## Supplementary Materials

The following are available online at https://www.mdpi.com/article/10.3390/biomedicines9050565/s1, Figure S1: Radiosynthesis of [^3^H]WC-DZ from the bromo-precursor WC-DZ-Br, Figure S2: Chiral analytical HPLC of talazoparib derivatives (a) **3a** (F), (b) **3b** (Br), (c) **3c** (I), Figure S3: Semi-preparative HPLC of [^18^F]**3a**, Figure S4: Chiral HPLC separation of [^18^F]talazoparib ([^18^F]**3a″**) and its enantiomer ([^18^F]**3a’**), Figure S5: Analytical chiral HPLC of [^18^F]talazoparib: (a) Animal dose; (b) Co-injection with authentic Talazoparib.
