# Supplementary material for: Radiosynthesis and Evaluation of Talazoparib and Its Derivatives as PARP-1-Targeting Agents
Source: Biomedicines. 2021 May 18;9(5):565. doi: 10.3390/biomedicines9050565 (PMC8157854; doi:10.3390/biomedicines9050565)
Supplement: Supplementary file 1 [file biomedicines-09-00565-s001.zip › biomedicines-1216809-supplementary.pdf]

*Supplementary Materials*

# Radiosynthesis and *in vitro* and *in vivo* evaluation of talazoparib and its derivatives as PARP-1 targeting agents for cancer

Dong Zhou, Huaping Chen, Cedric Mpoy, Sadia Afrin, Buck E. Rogers, Joel R. Garbow, John A. Katzenellenbogen, Jinbin Xu

## Table of contents

**Figure S1**. Radiosynthesis of [$^{3}$H]WC-DZ from the bromo-precursor WC-DZ-Br……….…………..……002

**Figure S2**. Chiral analytical HPLC of talazoparib derivatives (a) **3a** (F), (b) **3b** (Br), (c) **3c** (I)……....002

**Figure S4**. Chiral HPLC separation of [$^{18}$F]talazoparib ([$^{18}$F]**3a'**) and its enantiomer ([$^{18}$F]**3a''**)…...003

**Figure S5**. Analytical chiral HPLC of[$^{18}$F]talazoparib: (a) Animal dose; (b) Co-injection with authentic talazoparib……………………….....…..…………………………………………………………….…….004

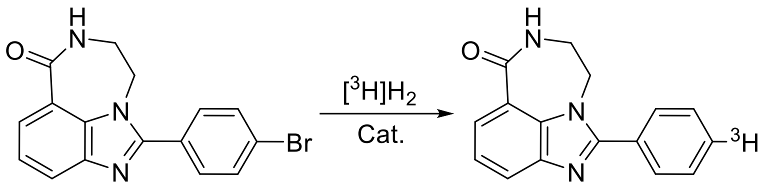


**Figure S1**. Radiosynthesis of [3H]WC-DZ from the bromo-precursor WC-DZ-Br

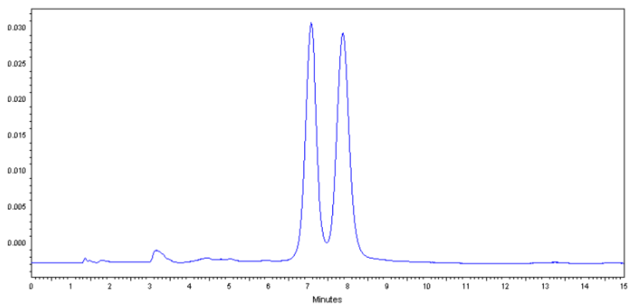


(a)

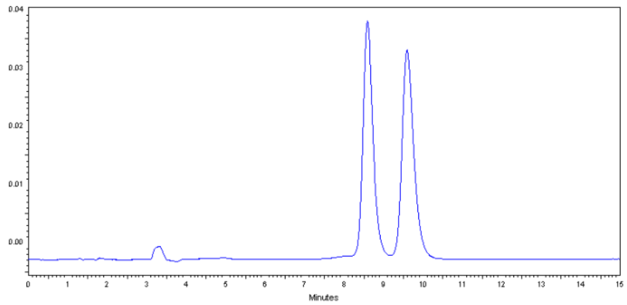


(b)

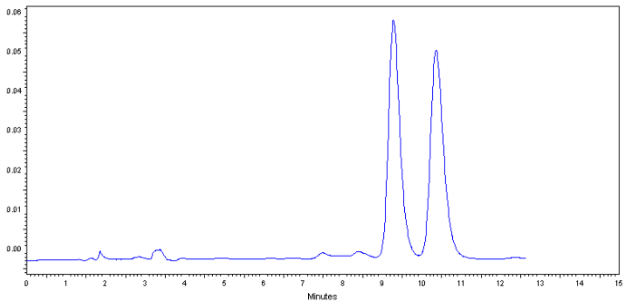


(c)

**Figure S2**. Chiral analytical HPLC of talazoparib derivatives (a) **3a** (F), (b) **3b** (Br), (c) **3c** (I). Column: Chirobiotech T; Mobile phase: 35% methanol/water; Flow rate: 1 mL/min; UV: 254 nm.

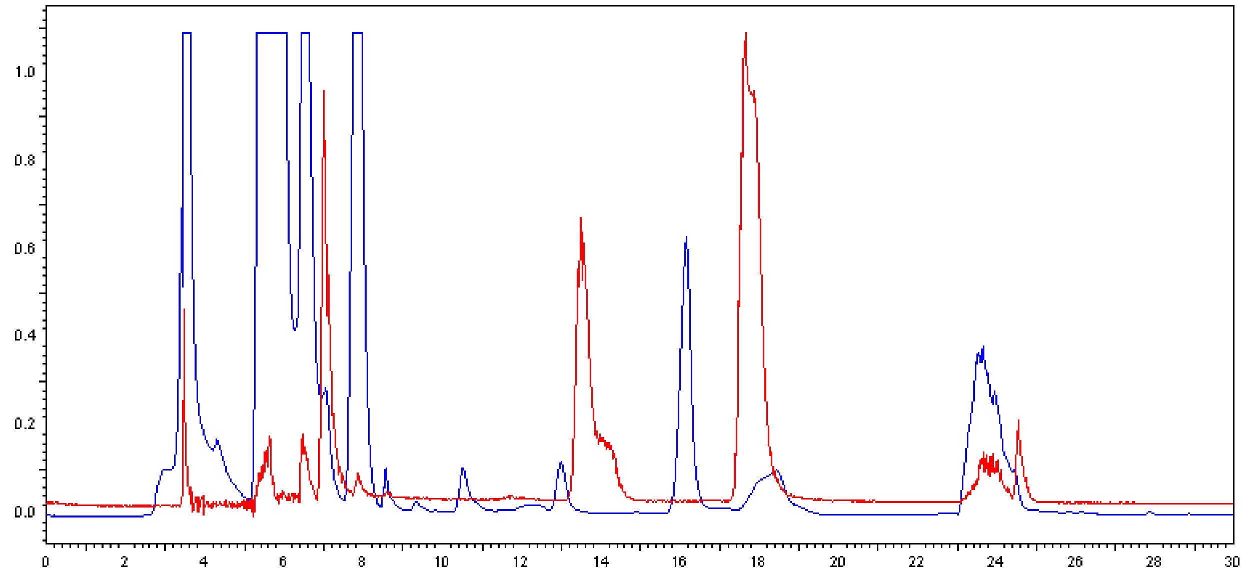


**Figure S3**. Semi-preparative HPLC of [18F]**3a**. [18F]**3a** was collected at 18 min. Blue: UV; Red: Radioactivity.

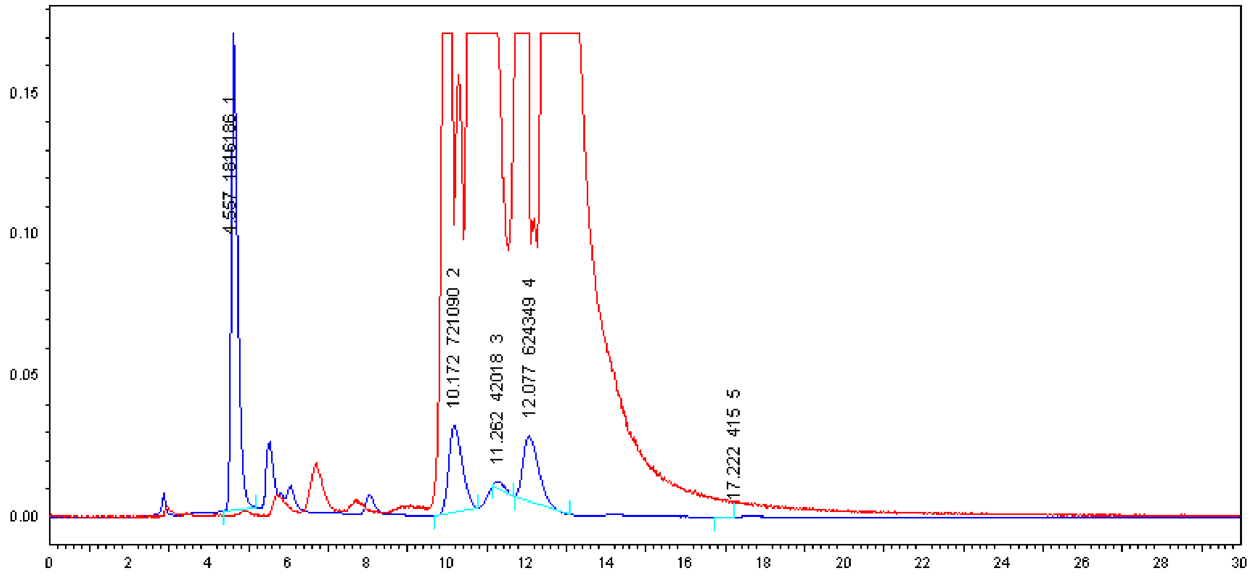


**Figure S4**. Chiral HPLC separation of [18F]talazoparib ([18F]**3a''**) and its enantiomer ([18F]**3a'**). [18F]Talazoparib ([18F]**3a''** was collected at 12.1 min. Blue: UV; Red: Radioactivity.

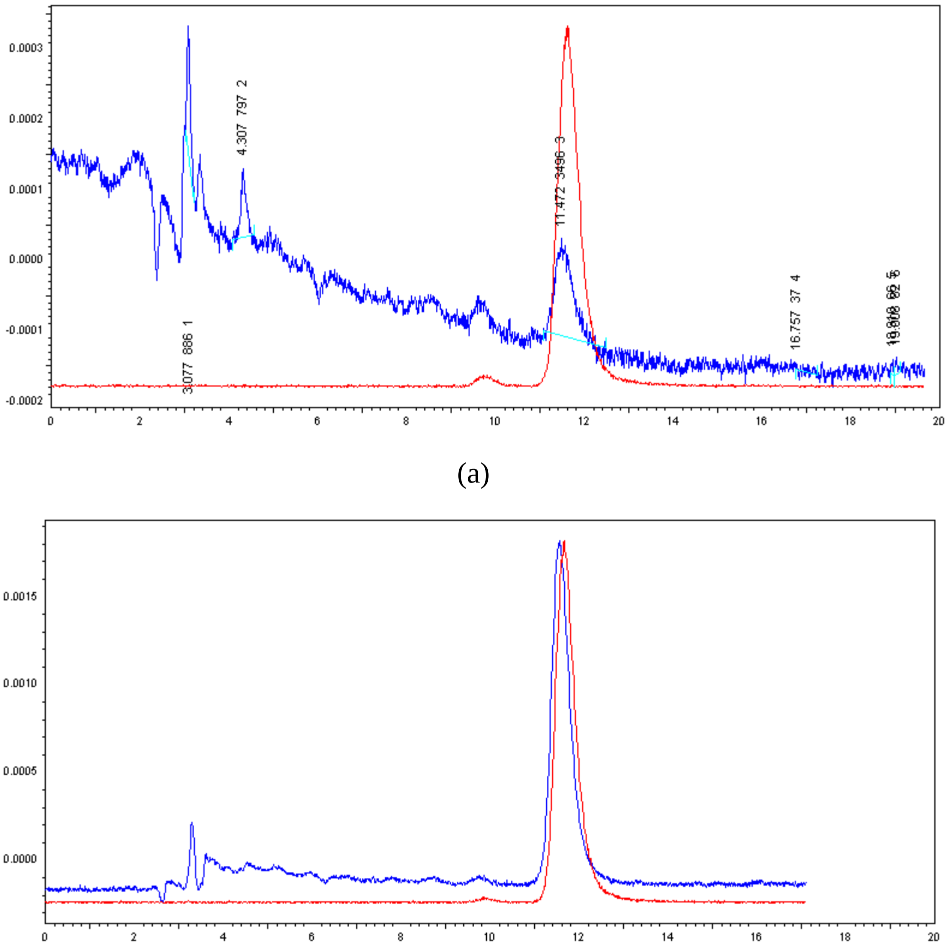


(a)

(b)

**Figure S5**. Analytical chiral HPLC of [$^{18}$F]talazoparib: (a) Animal dose; (b) Co-injection with authentic Talazoparib. Blue: UV; Red: Radioactivity.
